# Efficient similarity-based data clustering by optimal object to cluster reallocation

**DOI:** 10.1371/journal.pone.0197450

**Published:** 2018-06-01

**Authors:** Mathias Rossignol, Mathieu Lagrange, Arshia Cont

**Affiliations:** 1 Ircam, CNRS, Paris, France; 2 Ls2n, CRNS, Ecole Centrale Nantes, Nantes, France; Universitat Rovira i Virgili, SPAIN

## Abstract

We present an iterative flat hard clustering algorithm designed to operate on arbitrary similarity matrices, with the only constraint that these matrices be symmetrical. Although functionally very close to kernel k-means, our proposal performs a maximization of average intra-class similarity, instead of a squared distance minimization, in order to remain closer to the semantics of similarities. We show that this approach permits the relaxing of some conditions on usable affinity matrices like semi-positiveness, as well as opening possibilities for computational optimization required for large datasets. Systematic evaluation on a variety of data sets shows that compared with kernel k-means and the spectral clustering methods, the proposed approach gives equivalent or better performance, while running much faster. Most notably, it significantly reduces memory access, which makes it a good choice for large data collections. Material enabling the reproducibility of the results is made available online.

## Introduction

Clustering collections of objects into classes that bring together similar ones is probably the most common and intuitive tool used both by human cognition and artificial data analysis in an attempt to make that data organized, understandable, manageable. When the studied objects lend themselves to this kind of analysis, it is a powerful way to expose underlying organizations and approximate the data in such a way that the relationships between its members can be statistically understood and modeled. Given a description of objects, we first attempt to quantify which ones are “similar” from a given point of view, then group those *n* objects into *C* clusters, so that the similarity between objects within the same cluster is maximized. Finding the actual best possible partition of objects into clusters is, however, an NP-complete problem, intractable for useful datasets sizes. Many approaches have been proposed to yield an approximate solution: analytic, iterative, flat or hierarchical, agglomerative or divisive, soft or hard clustering algorithms, *etc.*, each with their strengths and weaknesses [1], performing better on some classes of problems than others [2, 3].

Iterative divisive hard clustering algorithms, usually perform well to identify high-level organization in large data collections in reasonable running time. For that reason, they are a sensible choice in many data mining situations, and constitute our focus in this paper. If the data lies in a vector space, *i.e.* an object can be described by a *m*-dimensional feature vector without significant loss of information, the seminal *k-means* algorithm [4] is probably the most efficient approach, since the explicit computation of the cluster centroids ensure both computational efficiency and scalability. This algorithm is based on the centroid model, and minimizes the intra cluster Euclidean distance. As shown by [5], any kind of Bregman divergence, such as the KL-divergence [6] or the Itakura-Saito divergence [7], may also be considered to develop such efficient clustering algorithms.

However, for many types of data, the projection of a representational problem into an vector space cannot be done without significant loss of descriptive efficiency. To reduce this loss, specifically tailored measures of similarity are considered. As a result, the input data for clustering is no longer a *n* × *m* matrix storing the *m*-dimensional vectors describing the objects, but a (usually symmetric) square matrix *S* of size *n* ×*n* which numerically encodes some sort of relationship between the objects. In this case, one has to resort to clustering algorithms based on connectivity models, since the cluster centroids cannot be explicitly computed.

Early attempts to solve this issue considered the k-medoids problem, where the goal is to find the *k* objects that maximize the average similarity with the other objects of their respective clusters, or *medoids*. The Partition Around Medoids (PAM) algorithm [8] solves the k-medoids problem but with a complexity of *O*(*k*(*n* − *k*)^2^)*i*, *n* being the number of objects and *i* number of iterations. Due to the high complexity and the low convergence rate, this algorithm cannot be applied to decent size datasets. In order to scale the approach, the Clustering LARge Applications (CLARA) algorithm [8] draws a sample of objects before running the PAM algorithm. This sampling operation is repeated several times and the most satisfying set of medoids is retained. In contrast, CLARANS [9] preserves the whole set of objects but cuts complexity by drawing a sample of neighbors in each search for the medoids.

Another classical approach to the issue is to run a variant of k-means that considers average intra-cluster similarity as the guiding criterion for object-to-class reallocation [10, Chapter 10.7]. This straightforward technique performs well in many cases, but the complexity of computing anew intra-cluster similarities at each iteration makes it impractical for large datasets.

Following work on kernel projection [11], that is, the fact that a nonlinear data transformation into some high dimensional feature space increases the probability of the linear separability of patterns within the transformed space, [12] introduced a kernel version of the K-means algorithm, whose input is a kernel matrix K that must be a Gram matrix, *i.e.* semi definite positive. [13] linked a weighted version of the kernel K-means objective to the popular spectral clustering [14], introducing an efficient way of solving the normalized cut objective [15].

The kernel k-means algorithm proves to be equally useful when considering arbitrary similarity problems if special care is taken to ensure definite positiveness of the input matrix [16]. This follows original algorithmic considerations where vector space data is projected into high dimensional spaces using a carefully chosen kernel function.

Despite such improvements, kernel k-means cannot be easily applied to large scale datasets without special treatments because of high algorithmic and memory access costs. [17] considered sampling of the input data, [18] considered block storing of the input matrix, and a pre-clustering approach [19] is considered by [20] with a coarsening and refining phases as respectively a pre- and post-treatment of the actual clustering phase.

We show in this paper that by using a semantically equivalent variant of the average intra-cluster similarity presented in [10, Chapter 10.7], it becomes possible to perform a computationally efficient greedy optimization [10, Chapter 10.8] with guaranteed convergence for arbitrary similarity measures. Although both of those techniques are well known, combining them is non trivial, and lead to a clustering algorithm, which we call k-averages with the following properties:

- input data can be arbitrary symmetric similarity matrices,
- it has fast and guaranteed convergence, with a number of object to clusters reallocations experimentally found to be roughly equal to the number of objects,
- it provides good scalability thanks to a reduced need for memory access, and
- on a collection of synthetic and natural test data, its results are equivalent to those of kernel k-means, and obtained in a fraction of its computing time, particularly when paged memory is required.

To summarize, the main contribution of the paper is to present a clustering algorithm:

- that can handle arbitrary affinity matrices, *i.e.* semi-positiveness is not a mandatory requirement for guaranteed convergence
- thanks to a carefully designed strategy to update the membership of objects to cluster, the algorithm is fast and memory efficient.

The remaining of the paper is organized as follows: Section 1 presents the kernel k-means objective function and the basic algorithm that minimizes this function, and Section 2 introduces the concepts behind the k-averages algorithm, followed by a detailed algorithmic description in Section 3. The complexity of the two algorithms in terms of arithmetic operations and memory access is then studied in Section 5. The above presented properties of the proposed k-averages algorithm are then validated on synthetic controlled data in Section 6 and on 43 datasets of time series issued from various sources in Section 7.

## 1 Kernel k-means

Since its introduction by [12], kernel k-means has been an algorithm of choice for flat data clustering with known number of clusters [16, 20]. It makes use of a mathematical technique known as the “kernel trick” to extend the classical k-means clustering algorithm [4] to criteria beyond simple euclidean distance proximity. Since it constitutes the closest point of comparison with our own work, we dedicate this section to its detailed presentation.

In the case of kernel k-means, the kernel trick allows us to consider that the k-means algorithm is operating in an unspecified, possibly very high-dimensional Euclidean space; but instead of specifying the properties of that space and the coordinates of objects, the equations governing the algorithm are modified so that everything can be computed knowing only the scalar products between points. The symmetrical matrix containing those scalar products is known as a kernel, noted K.

### 1.1 Kernel k-means objective function

In this section and the following, we shall adopt the following convention: *N* is the number of objects to cluster and *C* the number of clusters; *N*_*c*_ is the number of objects in cluster *c*, and *μ*_*c*_ is the centroid of that cluster. *z*_*cn*_ is the membership function, whose value is 1 if object *o*_*n*_ is in class *c*, 0 otherwise. In the folowing equations, *μ*_*c*_ and *o*_*n*_ are assumed to be column vectors of equal dimension.

Starting from the objective function minimized by the k-means algorithm, expressing the sum of squared distances of points to the centroids of their respective clusters:

$$\begin{array}{c} S=\sum_{c=1}^{C}\sum_{n=1}^{N}z_{cn}{\left(o_{n}-\mu_{c}\right)}^{⊤}\left(o_{n}-\mu_{c}\right) \end{array}$$

And using the definition of centroids as:

$$\begin{array}{c} \mu_{c}=\frac{1}{N_{c}}\sum_{n=1}^{N}z_{cn}o_{n} \end{array}$$

*S* can be developed and rewritten in a way that does not explicitly refer to the centroid positions, since those cannot be computed:

$$\begin{array}{c} S=\sum_{c=1}^{C}\sum_{n=1}^{N}z_{cn}Y_{cn} \end{array}$$

where

$$\begin{array}{ll} Y_{cn} & ={\left(o_{n}-\mu_{c}\right)}^{⊤}\left(o_{n}-\mu_{c}\right)\\ & =o_{n}o_{n}-2o_{n}^{⊤}\mu_{c}+\mu_{c}^{⊤}\mu_{c}\\ & =o_{n}^{⊤}o_{n}-2o_{n}^{⊤}\frac{1}{N_{c}}\sum_{i=1}^{N}z_{ci}o_{i}+{\left(\frac{1}{N_{c}}\sum_{i=1}^{N}z_{ci}o_{i}\right)}^{⊤}\left(\frac{1}{N_{c}}\sum_{i=1}^{N}z_{ci}o_{i}\right)\\ & =o_{n}^{⊤}o_{n}-\frac{2}{N_{c}}\sum_{i=1}^{N}z_{ci}o_{n}^{⊤}o_{i}+\frac{1}{N_{c}^{2}}\sum_{i=1}^{N}\sum_{j=1}^{N}z_{ki}z_{kj}o_{i}^{⊤}o_{j}\\ & =K_{nn}-\frac{2}{N_{c}}\sum_{i=1}^{N}z_{ci}K_{ni}+\frac{1}{N_{c}^{2}}\sum_{i=1}^{N}\sum_{j=1}^{N}z_{ki}z_{kj}K_{ij} \end{array}$$ (1)

Since the sum of *K*_*nn*_ over all points remains constant, and the sum of squared centroid norms (third, quadratic, term of Eq 1) is mostly bounded by the general geometry of the cloud of objects, we can see that minimizing this value implies maximizing the sum of the central terms, which are the average scalar products of points with other points belonging to the same class. Therefore, given a matrix gathering similarities between objects, if that matrix possesses the necessary properties to be considered as a kernel (positive semidefinitness), then the kernel k-means algorithm can be applied to it in order to create clusters that locally maximize the average intra-cluster similarity.

### 1.2 Algorithm

Finding the configuration that globally minimizes S (Eq 1.1) is an NP-complete problem. However, several approaches allow finding an acceptable approximation. We shall only focus here on the fastest and most popular, an iterative assignment / update procedure commonly referred to as the “k-means algorithm” [4], or as a discrete version of Lloyd’s algorithm, detailed in Algorithm 1.

**Algorithm 1:** Lloyd’s algorithm applied to minimizing the kernel k-means objective.

**Data:** number of objects *N*, number of classes *C*, kernel matrix K

**Result:** label vector *L* defining a partition of the objects into *C* classes

1 **Initialization:** fill L with random values in [1..*C*];

2 **while** *L is modified* **do**

3  **for** *n* ← 1 *to N* **do**

4   **for** *c* ← 1 *to C* **do**

5    Compute *Y*_*cn*_ following Eq 1 (note: *z*_*cn*_ = (*L*_*n*_ == *c*)?1: 0)

6   **end**

7   *L*_*n*_ = argmin_*c*_(*Y*_*cn*_);

8  **end**

9 **end**

The version given here is the most direct algorithmic translation of the mathematical foundations developed above, and as we shall see in section 5, it can easily become more efficient. Before that, we introduce our proposed k-averages algorithm.

## 2 Foundations of the k-averages algorithm

In our proposal, we adopt an alternative objective function which, unlike kernel k-means, does not rely on a geometric interpretation but an explicit account of the similarity matrix. The goal is to maximize the average intra-cluster similarity between points, a commonly used metric to evaluate clustering quality, and one whose computation is direct—linear in time.

Due to its simplicity, however, the objective function cannot be simply “plugged into” the standard kernel k-means algorithm: it lacks the geometric requisites to ensure convergence. We must therefore propose a specifically tailored algorithmic framework to exploit it: first, we show here that it is possible to easily compute the impact on the global objective function of moving a single point from one class to another; this allows us to develop a greedy optimization algorithm taking advantage of that formula.

### 2.1 Conventions and objective function

In addition to the notations presented above, we index here the set of elements belonging to a given cluster *c*_*k*_ as $c_{k}=\{o_{k1},\ldots ,o_{kN_{k}}\}$. For simplicity, we omit the first index and note $c=\{o_{1},\ldots ,o_{N_{c}}\}$ when considering a single class.

The similarity between objects shall be written *s*(*o*_*i*_, *o*_*j*_). We extend the notation *s* to the *similarity of an object to a class* defined as the average similarity of an object with all objects of the class. *s*(*o*, *c*) accepts two definitions, depending on whether or not *o* is a member of *c*:

If *o* ∉ *c*,

$$\begin{array}{c} s(o,c)=\frac{1}{N_{c}}\sum_{i=1}^{n_{c}}s(o,o_{i}) \end{array}$$ (2)

If *o* ∈ *c*, then necessarily ∃*i* ∣ *o* = *o*_*i*_

$$\begin{array}{c} s(o,c)=s(o_{i},c)=\frac{1}{N_{c}-1}\sum_{j=1\ldots n_{c},j\neq i}s(o_{i},o_{j}) \end{array}$$ (3)

Let us call the “quality” of a class the average intra-class object-to-object similarity, and write it Q:

$$\begin{array}{c} Q(c)=\frac{1}{N_{c}}\sum_{i=1}^{n_{c}}s(o_{i},c) \end{array}$$ (4)

In our framework, we do not explicitly refer to class centroids, preferring to directly consider averages of similarity values between individuals within clusters. Using the notations above, we define our objective function as the average class quality, normalized with class sizes:

$$\begin{array}{c} O=\frac{1}{N}\sum_{i=1}^{C}N_{i}Q\left(c_{i}\right) \end{array}$$ (5)

Since, informally, our goal is to bring together objects that share high similarity, a first idea would be to simply repeatedly move each object to the class with whose members it has the highest average similarity. This is what we call the “naive k-averages” algorithm.

### 2.2 Naive k-averages algorithm

Algorithm 2 presents a method that simply moves each object to the class with which it has the highest average similarity, until convergence is reached. The algorithm is straightforward and simple; however, experiments show that while it can often produce interesting results, it also sometimes cannot reach convergence because the decision to move an object to a different cluster is taken without considering the impact of the move on the quality of the source cluster.

**Algorithm 2:** The naive k-averages algorithm.

**Data:** number of objects *N*, number of classes *C*, similarity matrix S

**Result:** label vector *L* defining a partition of the objects into *C* classes

1 **Initialization:** Fill L with random values in [1..*C*];

2 Compute initial object-class similarities *S* following Eq 3 or Eq 2;

3 **while** *L is modified* **do**

4  **for** *i* ← 1 *to N* **do**

5   previousClass ← *L*_*i*_;

6   nextClass ← argmin_*k*_ *S*(*i*, *k*) **if** *nextClass* ≠ *previousClass* **then**

7    *L*_*i*_ ← nextClass;

8    **for** *j* ← 1 *to N* **do**

9     Update *S*(*j*, *nextClass*) and *S*(*j*, *previousClass*)

10    **end**

11   **end**

12  **end**

13 **end**

To ensure convergence, we need to compute the impact on the objective function of moving one object from one class to another. Using such formulation and performing only reallocation that have a positive impact, the convergence of such an iterative algorithm is guaranteed.

### 2.3 Impact of object reallocation on class quality

Considering a class *c*, let us develop the expression of Q(c) into a more useful form. Since all objects are in *c*, we use the formula in (Eq 3) to get:

$$\begin{array}{ll} Q\left(c\right) & =\frac{1}{N_{c}}\sum_{i=1}^{N_{c}}\frac{1}{N_{c}-1}\sum_{\begin{array}{c} j=1\ldots N_{c}\\ j\neq i \end{array}}s\left(o_{i},o_{j}\right)\\ & =\frac{1}{N_{c}\left(N_{c}-1\right)}\sum_{i=1}^{N_{c}}\sum_{\begin{array}{c} j=1\ldots N_{c}\\ j\neq i \end{array}}s\left(o_{i},o_{j}\right) \end{array}$$

Using the assumption that the similarity matrix is symmetrical, we can reach:

$$\begin{array}{c} Q(c)=\frac{2}{N_{c}\left(N_{c}-1\right)}\sum_{i=2}^{N_{c}}\sum_{j=1}^{i-1}s(o_{i},o_{j}) \end{array}$$ (6)

For future use and given the importance of the above transformation, we define:

$$\begin{array}{c} \Sigma \left(c\right)=\sum_{i=2}^{N_{c}}\sum_{j=1}^{i-1}s(o_{i},o_{j}) \end{array}$$

Thus:

Q(c)=2Nc(Nc-1)Σ(c)XXandXXΣ(c)=Nc(Nc-1)Q(c)2

#### 2.3.1 Removing an object from a class

Assuming that *o* ∈ *c*, necessarily ∃*i* ∣ *o* = *o*_*i*_. Since the numbering of objects is arbitrary, we can first simplify the following equation by considering that $o=o_{N_{c}}$, in order to reach a formula that is independent from that numbering.

$$\begin{array}{cc} Q(c\backslash o_{N_{c}}) & =\frac{2}{\left(N_{c}-1\right)\left(N_{c}-2\right)}\sum_{i=2}^{N_{c}-1}\sum_{j=1}^{i-1}s(o_{i},o_{j})\\ & =\frac{2}{\left(N_{c}-1\right)\left(N_{c}-2\right)}[\Sigma \left(c\right)-\sum_{j=1}^{N_{c}-1}s(o_{N_{c}},o_{j})]\\ & =\frac{2}{\left(N_{c}-1\right)\left(N_{c}-2\right)}[\Sigma \left(c\right)-\left(N_{c}-1\right)s(o_{N_{c}},c)]\\ & =\frac{2N_{c}\left(N_{c}-1\right)Q\left(c\right)}{2\left(N_{c}-1\right)\left(N_{c}-2\right)}-\frac{2\left(N_{c}-1\right)s(o_{N_{c}},c)}{\left(N_{c}-1\right)\left(N_{c}-2\right)}\\ & =\frac{N_{c}Q\left(c\right)-2s(o_{N_{c}},c)}{N_{c}-2} \end{array}$$

The quality of a class after removal of an object is thus:

$$\begin{array}{c} Q(c\backslash o)=\frac{N_{c}Q\left(c\right)-2s(o,c)}{N_{c}-2} \end{array}$$ (7)

And the change in quality from its previous value:

$$\begin{array}{cc} Q(c\backslash o)-Q(c) & =\frac{N_{c}Q\left(c\right)-\left(N_{c}-2\right)Q\left(c\right)-2s(o,c)}{N_{c}-2}\\ & =\frac{2(Q\left(c\right)-s(o,c))}{N_{c}-2} \end{array}$$ (8)

#### 2.3.2 Adding an object to a class

Assuming that *o* ∉ *c*, we can similarly to what has been done previously (numbering is arbitrary) consider for the sake of simplicity that *o* becomes *o*_*N*_*c*_+1_ in the modified class *c*. Following a path similar to above, we get:

$$\begin{array}{cc} Q(c\cup o_{N_{c}+1}) & =\frac{2}{N_{c}\left(N_{c}+1\right)}\sum_{i=2}^{N_{c}+1}\sum_{j=1}^{i-1}s(o_{i},o_{j})\\ & =\frac{2}{N_{c}\left(N_{c}+1\right)}[\Sigma \left(c\right)+N_{c}s(o_{N_{c}+1},c)]\\ & =\frac{\left(N_{c}-1\right)Q\left(c\right)+2s(o_{N_{c}+1},c)}{N_{c}+1} \end{array}$$

The quality of a class *c* after adding an object *o* is thus:

$$\begin{array}{c} Q(c\cup o)=\frac{\left(N_{c}-1\right)Q\left(c\right)+2s(o,c)}{N_{c}+1} \end{array}$$ (9)

And the change in quality from its previous value:

$$\begin{array}{c} Q(c\cup o)-Q(c)=\frac{2(s(o,c)-Q\left(c\right))}{N_{c}+1} \end{array}$$ (10)

### 2.4 Impact of object reallocation on the global objective function

When moving an object *o* from class *c*_*s*_ (“source”), to whom it belongs, to a distinct class *c*_*t*_ (“target”), (*N*_*s*_ − 1) objects are affected by the variation in (8), and *N*_*t*_ are affected by that in (10); in addition, one object *o* moves from a class whose quality is $Q(c_{s})$ to one whose quality is $Q(c_{t}\cup o)$, as expressed by Eq 9, which leads to an impact of moving object *o* from class *c*_*s*_ to class *c*_*t*_ wich can be computed as follows:

$$\begin{array}{c} \begin{array}{cc} \delta_{o}\left(c_{s},c_{t}\right)= & \frac{2N_{t}(s(o,c_{t})-Q\left(c_{t}\right))}{N_{t}+1}+\\ & \frac{2\left(N_{s}-1\right)(Q\left(c_{s}\right)-s(o,c_{s}))}{N_{s}-2}+\\ & \frac{\left(N_{t}-1\right)Q\left(c_{t}\right)+2s(o,c_{t})}{N_{t}+1}-Q\left(c_{s}\right) \end{array} \end{array}$$ (11)

As can be seen, computing this impact is a fixed-cost operation. We can therefore use the formula as the basis for an efficient iterative algorithm.

## 3 K-averages algorithm

**Algorithm 3:** The K-averages algorithm.

**Data:** number of objects *N*, number of classes *C*, similarity matrix S

**Result:** label vector *L* defining a partition of the objects into *C* classes

1 **Initialization:** Fill L with random values in [1..*C*];

2 Compute initial object-class similarities *S* following Eq 3 or Eq 2;

3 Compute initial class qualities Q following Eq 6;

4 **while** *L is modified* **do**

5  **for** *i* ← 1 *to N* **do**

6   previousClass ← *L*_*i*_;

7   nextClass ← argmax_*k*_ *δ*_*i*_(previousClass, *k*) (following the definition of *δ* in Eq 11);

8   **if** *nextClass* ≠ *previousClass* **then**

9    *L*_*i*_ ← nextClass;

10    Update $Q_{\text{previousClass}}$ following Eq 7;

11    Update $Q_{\text{nextClass}}$ following Eq 9;

12    **for** *j* ← 1 *to N* **do**

13     Update *S*(*j*, *nextClass*) and *S*(*j*, *previousClass*)

14     following Eq 12;

15    **end**

16   **end**

17  **end**

18 **end**

Our approach does not allow us to benefit, like kernel k-means, from the convergence guarantee brought by the geometric foundation of k-means. In consequence, we cannot apply a “batch” approach where at each iteration all elements are moved to their new class, and all distances (or similarities) are computed at once. To guarantee convergence, we must update the class properties for the two modified classes (source and destination), as well as recompute the average class-object similarities for them for each considered object, after finding its ideal new class. This is the principle of the “greedy” k-means algorithm [10, Chapter10.8], but whereas for k-means that approach increases complexity (and even more so for kernel k-means), in this case it leads to a much improved computational performance.

Indeed, at a first glance, dynamically updating objectives as a result of object reallocation might seem to have negative performance impact. However, our simple non-quadratic updates make such dynamic changes easily tractable. New class qualities are thus given by Eqs 7 and 9, and new object-class similarities can be computed by:

$$\begin{array}{cc} s(i,c_{s}\left(t+1\right)) & =\frac{N_{s}\left(t\right).s\left(i,c_{s}\left(t\right)\right)+s\left(i,n\right)}{N_{s}\left(t\right)+1}\\ s(i,c_{t}\left(t+1\right)) & =\frac{N_{t}\left(t\right).s\left(i,c_{s}\left(t\right)\right)-s\left(i,n\right)}{N_{t}\left(t\right)-1} \end{array}$$ (12)

where *i* is any object index, *n* is the recently reallocated object, *c*_*s*_ the “source” class that object *i* was removed from, and *c*_*t*_ the “target” class that object *n* was added to.

The full description of k-averages is given in Algorithm 3.

## 4 Convergence

The kernel k-means algorithm ensures convergence if the similarity matrix is semi-definite positive. The k-averages algorithm relaxes this constraint by only requiring symmetricity of the similarity matrix to ensure convergence.

An algorithm is guaranteed to converge if its successive iterations can be tied to a strictly monotonous and bounded quantity. For the k-averages algorithm, this quantity is the objective function itself, as we now show.

Thanks to the rewriting of the class quality function done in Eq 6, which only requires the similarity matrix to be symmetrical, we can directly define the allocation decision criterion *δ* (Eq 11) to be the change in the global objective function O (Eq 5) implied by reallocating an object to a new class. It follows that, as long as reallocations are only performed when *δ* > 0, O is strictly increasing throughout the execution of the algorithm.

Moreover, O, defined as a weighted average of the average intra-class similarities for the produced clusters, can be proven to never exceed the maximal similarity between two objects.

Indeed, the average similarity of an object to other members of its class, expressed as *s*(*o*, *c*) in Eq 2, is an average of similarities, and therefore lower than or equal to their maximum value. Similarly, the quality Q of a class, defined as the average of object to class similarities (Eq 4), is inferior to the maximum value of *s*(*o*, *c*), and therefore to the maximum similarity between objects. Finally, O, a weighted average of Q values, is, once again, inferior to their maximum value.

The objective function O is thus upper-bounded, and increases at each iteration of the outer loop of Algorithm 3; which guarantees its convergence.

## 5 Complexity analysis

In this section, we study the complexity of the two approaches presented above, first from the point of view of raw complexity, second by focusing on memory access.

### 5.1 Computational complexity

#### 5.1.1 Kernel k-means

As can be seen in Algorithm 1, the operation on line 5 is the most costly part of the algorithm: for each object *n* and class *c*, at each iteration, it is necessary to compute *Y*_*cn*_ from Eq 1—an *O*(*N*^2^) operation in itself, per object. The impossibility of simply computing the distances to a known centroid as done in the k-means algorithm leads to a much higher complexity for the kernel k-means algorithm, globally *O*(*N*^3^) per iteration, independent of how many objects are moved for that iteration.

It is however possible to improve the performance of kernel k-means by noting than in Eq 1, the third term of the equation, which has the highest complexity, is only dependent on class definitions and not on the considered object. We can therefore rewrite Eq 1 as:

$$\begin{array}{ccc} Y_{cn} & = & K_{nn}-\frac{2}{N_{c}}\sum_{i=1}^{N}z_{ci}K_{ni}+M_{c} \end{array}$$ (13)

where

$$\begin{array}{ccc} M_{c} & = & \frac{1}{N_{c}^{2}}\sum_{i=1}^{N}\sum_{j=1}^{N}z_{ki}z_{kj}K_{ij} \end{array}$$ (14)

Algorithm 1 thus becomes Algorithm 4, where the values of *M*_*c*_ are computed once at the beginning of each loop (line 4) then reused on line 8, thus reducing the overall complexity to *O*(*n*^2^) per iteration. This optimized version of kernel k-means is the one we shall consider for performance comparison in the remainder of this article.

**Algorithm 4:** Lloyd’s algorithm applied to minimizing the kernel k-means objective, optimized version.

**Data:** number of objects *N*, number of classes *C*, kernel matrix K

**Result:** label vector *L* defining a partition of the objects into *C* classes

1 **Initialization:** fill L with random values in [1..*C*];

2 **while** *L is modified* **do**

3  **for** *c* ← 1 *to C* **do**

4   Compute *M*_*c*_ following Eq 14

5  **end**

6  **for** *n* ← 1 *to N* **do**

7   **for** *c* ← 1 *to C* **do**

8    Compute *Y*_*cn*_ following Eq 13 (note: *z*_*cn*_ = (*L*_*n*_ == *c*)?1: 0)

9   **end**

10   *L*_*n*_ = argmin_*c*_(*Y*_*cn*_);

11  **end**

12 **end**

#### 5.1.2 K-averages

For the k-averages method presented as Algorithm 3, the complexity of each iteration is

- *O*(*NC*) corresponding to the best class search at line 7
- *O*(*NM*) corresponding to the object-to-class similarity update at line 13, where *M* is the number of objects moved at a given iteration.

**Fig 1:**
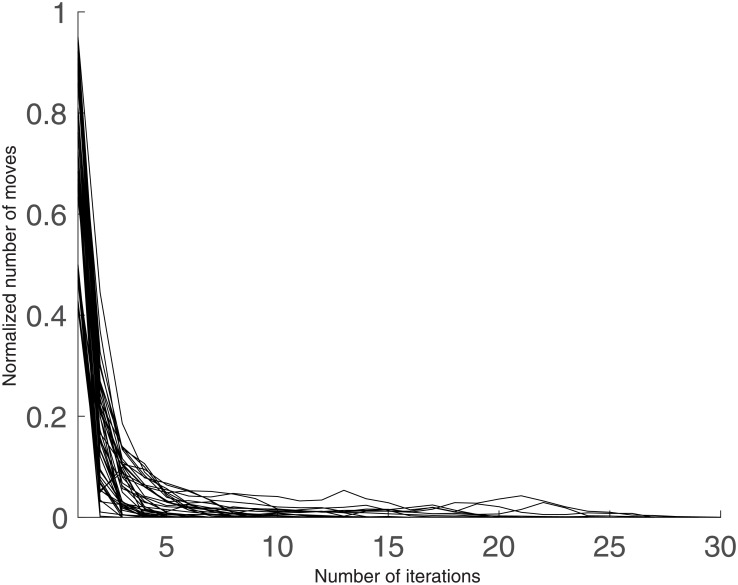
Number of moved objects per iteration when clustering a variety of datasets with the k-averages algorithm, normalized by the total number of objects to cluster. The datasets used to create this figure are the real-life time series data that we employ for experimental validation evaluated under the Dynamic Time Warping (DTW) similarity measure, *cf.* Section 7.

In the worst case scenario, *M* = *N*, and the complexity for one iteration of the algorithm remains the same as for the optimized kernel k-means algorithm, *O*(*N*^2^). In practice, however, as can be seen on Fig 1, the number of objects moving from one class to another decreases sharply after the first iteration, meaning that the complexity of one iteration becomes quickly much lower than *O*(*N*^2^). Thus, while the first iteration of k-averages has a similar complexity with kernel k-means, the overall cost of a typical run of the algorithm (from 10 to 50 iterations) is much lower.

**Fig 2:**
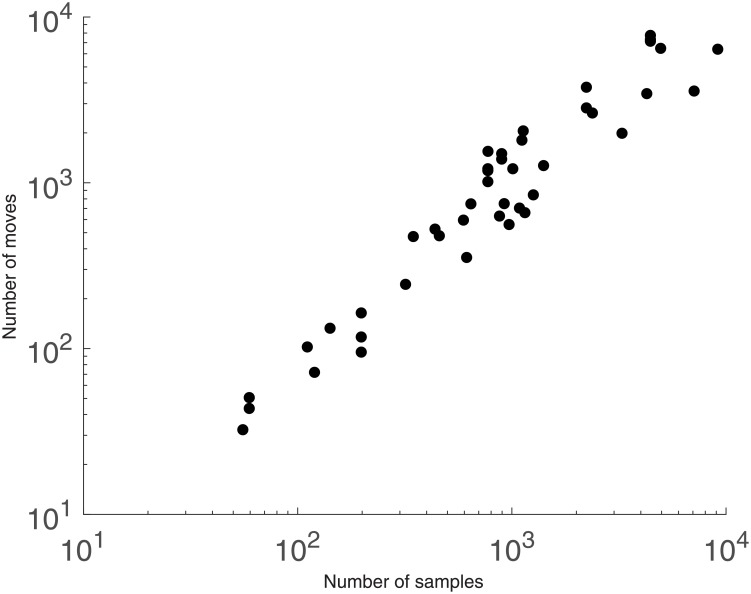
Total number of object reallocations over a run of the k-averages algorithm, plotted against the number of objects to be clustered. The datasets used to create this figure are the real-life time series data that we employ for experimental validation, *cf.* Section 7.

To go further in this analysis, we display on Fig 2 the total number of object reallocation over a full run of the k-averages algorithm for several datatsets. The datasets used to create this figure are the real-life time series data that we employ for experimental validation evaluated under the Dynamic Time Warping (DTW) similarity measure, *cf.* Section 7. As can be seen, the correlation is roughly linear with the number of objects to cluster. In fact, the number of reallocations is roughly equal to the number of objects to cluster, which allows us to reach for k-averages a (statistical) total complexity of *O*(*N*^2^), instead of *O*(*N*^2^) per iteration.

### 5.2 Memory access

The lowered computational costs is also accompanied by a decrease in memory access: as can be seen from Eq 12, in order to compute the new object-to-class similarities after moving an object *n*, only line *n* of the similarity matrix needs to be read. For the remaining of the algorithm, only the (much smaller) object-to-class similarity matrix is used. By contrast, in the case of kernel k-means, the computation of *M*_*c*_ values at each iteration require that the whole similarity matrix be read, which can be a serious performance bottleneck in the case of large object collections.

Moreover, the similarity update function of k-averages, by reading one line of the matrix at a time, presents good data locality properties, which make it play well with standard memory paging strategies.

To illustrate and confirm the theoretical complexity computed here, the next section proposes some performance figures measured on controlled datasets.

## 6 Validation

In order to reliably compare the clustering quality and execution speed between the two approaches, we have written plain C implementations of Algorithms 4 and 3, with minimal operational overhead: reading the similarity matrix from a binary file where all matrix values are stored sequentially in standard reading order, line by line, and writing out the result of the clustering as a label text file. Both implementations use reasonably efficient code, but without advanced optimizations or parallel processing.

The figures presented in this section were obtained on synthetic datasets, created in order to give precise control on the features of the analyzed data: for *n* points split between *C* classes, *C* centroids are generated at random in two dimensional space, and point coordinates are generated following a Gaussian distribution around class centroids. In addition to the numbers of objects and classes, the variance of Gaussian distributions are adjusted to modulate how clearly separable clusters are. Similarities are computed as inverse Euclidean distances between points.

### 6.1 Reproducibility

In order to ease reproducibility of the results, the data is taken from a public repository of several benchmark datasets used in academia [21] and the code of the proposed method as well as the experimental code used for generated the figures is publicly available: https://github.com/mathieulagrange/kaveragePaper.

### 6.2 Clustering performance

Several metrics are available to evaluate the performance of a clustering algorithm. The one closest to the actual target application is the raw accuracy, that is the average number of items labeled correctly after an alignment phase of the estimated labeling with the reference [22].

Another metric of choice is the Normalized Mutual Information (NMI) criterion. Based on information theoretic principles, it measures the amount of statistical information shared by the random variables representing the predicted cluster distribution and the reference class distribution of the data points. If *P* is the random variable denoting the cluster assignments of the points, and *C* is the random variable denoting the underlying class labels on the points then the NMI measure is defined as:

$$\begin{array}{c} \text{NMI}=\frac{2I(C;K)}{H(C)+H(K)} \end{array}$$

where *I*(*X*; *Y*) = *H*(*X*) − *H*(*X*|*Y*) is the mutual information between the random variables *X* and *Y*, *H*(*X*) is the Shannon entropy of *X*, and *H*(*X*|*Y*) is the conditional entropy of *X* given *Y*. Thanks to the normalization, the metric stays between 0 and 1, 1 indicating a perfect match, and can be used to compare clustering with different numbers of clusters. Interestingly, random prediction gives an NMI close to 0, whereas the accuracy of a random prediction on a balanced bi-class problem is as high as 50%.

In this paper, for simplicity sake, only the NMI is considered for validations. However, we found that most statements hereafter in terms of performance ranking of the different algorithms still hold while considering the accuracy metric as reference.

**Fig 3:**
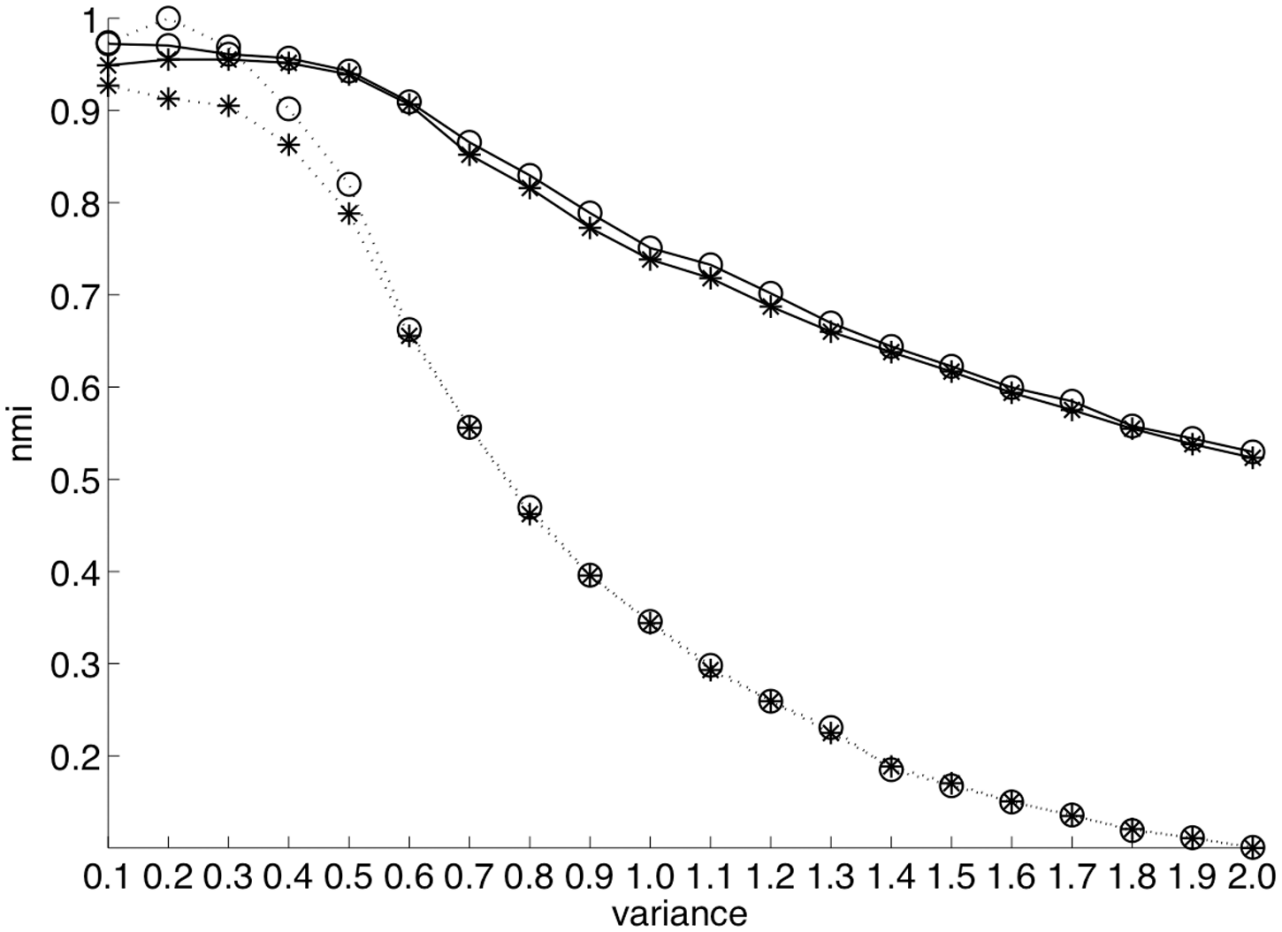
NMI of kernel k-means (*) and k-averages (o) clustering relative to ground truth as a function of the “spread” of those classes for synthetic data sets of 5 and 40 classes, displayed in dashed and solid lines, respectively.

Fig 3 presents the quality of clusterings obtained using kernel k-means and k-averages on two series of datasets: one featuring 5 classes, the other 40 classes. On the x-axis is the variance of the Gaussian distribution used to generate the point cloud for each class: the higher that value, the more the classes are spread out and overlap each other, thus making the clustering harder.

The question of choosing the proper number of clusters for a given dataset without *a priori* is a well known and hard problem, and beyond the scope of this article. Therefore, for the purpose of evaluation, clustering is done by requesting a number of clusters equal to the actual number of classes in the dataset. In order to obtain stable and reliable figures, clustering is repeated 500 times with varying initial conditions, *i.e.* the initial assignment of points to clusters is randomly determined, and only the average performance is given. For fairness of comparison, each algorithm is run with the exact same initial assignments.

As can be seen on the figure, in the case of a 5-class problem, k-averages outperforms kernel k-means in the “easy” cases (low class spread), before converging to equivalent results. For the more complex 40-class datasets, k-averages consistently yields a better result than kernel k-means, especially for higher values of the variance. The lower values of NMI for 5-class experiments is in fact an artifact introduced by the normalization of NMI, and is not important here; we only focus, for each series of experiments, on the relative performances of kernel k-means and k-averages.

### 6.3 Time efficiency

**Fig 4:**
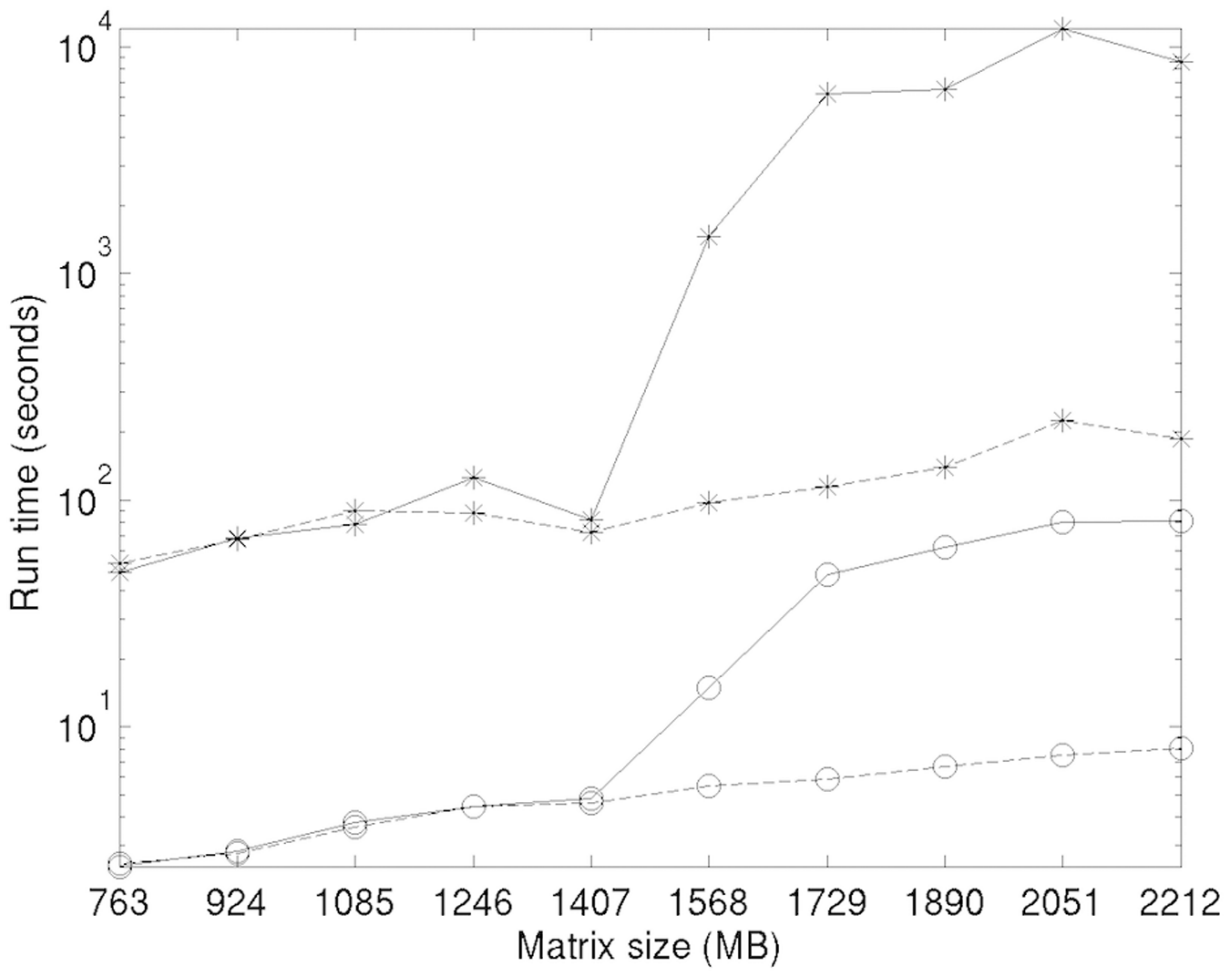
Average computation time of the kernel k-means (*) and kaverages (o) algorithms on computers with 2 GB (solid line) and 32 GB (dashed line) of RAM, respectively. The “running time” axis follows a logarithmic scale.

Fig 4 shows the average time spent by kernel k-means and k-averages to cluster synthetic datasets or varying sizes. As previously, initial conditions on each run are identical for both algorithms. The reported run time is the one measured on a 64 bits Intel^®^ Core^™^ i7 runnning at 3.6 GHz with 32 Gb of RAM and standard Hard Disk Drive (HDD) operated by standard Linux distribution. For results with 2 GB of RAM, the same machine is used with a memory limitation specified to the kernel at boot time.

These figures confirm the theoretical complexity analysis presented in Section 5: k-averages runs at least 20 times faster on average than kernel k-means in ordinary conditions, when available memory is not an issue. When the matrix size exceeds what can be stored in RAM and the system has to resort to paged memory, as in the presented experiments when the matrix reaches about 1500MB, both algorithms suffer from a clear performance hit; however, kernel k-means is much more affected, and the difference becomes even more important: with a 2000MB similarity matrix on a memory-limited computer, k-averages runs about 100 times faster than kernel k-means.

Having established the interest of our proposed method relative to kernel k-means on synthetic object collections, we now proceed to a thorough evaluation on real data.

## 7 Experiments

In order to demonstrate the usefulness of k-averages when dealing with real data, we have chosen to focus on the clustering of time series as the evaluation task. Time series, even though represented as vectors and therefore suitable for any kinds of norm-based clustering, are best compared with elastic measures [23, 24], partly due to their varying length. The Dynamic Time Warping (DTW) measure is an elastic measure widely used in many areas since its introduction for spoken word detection [25] and has never been challenged for time series mining [26, 27].

Effective clustering of time series using the DTW measure requires similarity based algorithms such as the k-averages algorithm. With some care, kernel based algorithm can also be considered provided that the resulting similarity matrix is converted into a kernel, *i.e.* the matrix is forced to be semi definite positive, *i.e.* to be a Gram matrix [28] in order to guarantee convergence.

### 7.1 Datasets

To compare quality of clusterings obtained by the considered algorithms, we consider a large collection of 43 time series datasets made publicly available by many laboratories worldwide and compiled by Prof. Keogh. Thus, while all the experiments presented here are performed on time series (chosen for being a good example of a data type requiring similarity-based clustering, as opposed to a simple Euclidean approach), the great variety in the sources and semantics of said series (bio-informatics, linguistics, astronomy, gesture modeling, chemistry…) gives this validation a wide foundation. Statistics about the morphology of those datasets can be found in Table 1 and summarized in Table 2.

**Table 1:** Description of the time series datasets used for evaluation.

name	number of classes	number of samples
50words	50	905
Adiac	37	781
Beef	5	60
CBF	3	930
ChlorineConcentration	3	4307
CinC_ECG_torso	4	1420
Coffee	2	56
Cricket_X	12	780
Cricket_Y	12	780
Cricket_Z	12	780
DiatomSizeReduction	4	322
ECG200	2	200
ECGFiveDays	2	884
FaceAll	14	2250
FaceFour	4	112
FacesUCR	14	2250
Gun_Point	2	200
Haptics	5	463
InlineSkate	7	650
ItalyPowerDemand	2	1096
Lighting2	2	121
Lighting7	7	143
MALLAT	8	2400
MedicalImages	10	1141
MoteStrain	2	1272
OSULeaf	6	442
OliveOil	4	60
SonyAIBORobotSurface	2	621
SonyAIBORobotSurfaceII	2	980
StarLightCurves	3	9236
SwedishLeaf	15	1125
Symbols	6	1020
Trace	4	200
TwoLeadECG	2	1162
Two_Patterns	4	5000
WordsSynonyms	25	905
fish	7	350
synthetic_control	6	600
uWaveGestureLibrary_X	8	4478
uWaveGestureLibrary_Y	8	4478
uWaveGestureLibrary_Z	8	4478
wafer	2	7164
yoga	2	3300

**Table 2:** Statistics of the datasets. The length of the times series is expressed in samples.

	min	average ± variance	max
number of classes	2	8 ± 9	50
number of time series	56	1626 ± 2023	9236
time series length	24	372 ± 400	1882

### 7.2 Methods

Three algorithms are considered: the spectral clustering [14] approach as a high complexity reference, the kernel k-means algorithm implemented as described in Section 1 and the proposed k-averages algorithm. The spectral clustering algorithm tested here uses the normalization proposed by Jordan and Weiss [29]. This normalization is chosen over no normalization and the Shi and Malik one [15] as it is found to be the best performing in terms of average NMI over all the datasets. The implementation is done using the Matlab programming language. Even though a C implementation would probably be more efficient, we believe that the gain would be low as the main computational load is the diagonalization of the similarity matrix and the k-means clustering of the eigenvectors which are both efficient builtins Matlab functions. The kernel k-means is implemented both in Matlab using the implementation provided by Mo Chen (vailable at: https://fr.mathworks.com/matlabcentral/fileexchange/26182-kernel-kmeans) and in the C programming language following Algorithm 1. The k-averages method is implemented in C following Algorithm 3.

### 7.3 Evaluation protocol

For each dataset, since we perform clustering, and not supervised learning, the training and testing data are joined together. DTW similarities are computed using the implementation provided by Prof. Ellis (available at: http://www.ee.columbia.edu/~dpwe/resources/matlab/dtw) with default parameters.

As in our previous experiments with synthetic data, we choose here the normalized mutual information (NMI) as the measure of clustering quality; clustering is done by requesting a number of clusters equal to the actual number of classes in the dataset, and repeated 200 times with varying initial conditions, each algorithm being run with the exact same initial assignments. For the 200 clusterings thus produced, we compute the NMI between them and the ground truth clustering. Average and standard deviation statistics are then computed.

### 7.4 Clustering performance

For ease of readability and comparison, the presented results are split into 3 tables. Table 3 lists the results obtained on bi-class datasets, *i.e.* the datasets annotated in terms of presence or absence of a given property; Table 4 concerns the datasets with a small number of classes (from 3 to 7); and Table 5 focuses the datasets with a larger number of classes (from 8 to 50).

**Table 3:** NMI (in percents) of clusterings by kernel k-means and k-averages for bi-class datasets.

	spectral clustering	kernel k-means	k-averages
Coffee	3.4 ±0.0	6.9 ±4.1	**7.8 ±3.6**
ECG200	9.9 ±0.0	**14.8 ±1.0**	14.6 ±0.0
ECGFiveDays	0.1 ±0.0	**3.3 ±0.3**	2.5 ±0.0
Gun_Point	**14.8 ±1.3**	0.0 ±0.0	0.0 ±0.0
ItalyPowerDemand	**1.1**	0.9	0.9
Lighting2	4.1 ±0.3	**4.9 ±3.7**	4.3 ±4.2
MoteStrain	0.0 ±0.0	**48.9 ±0.6**	48.8 ±0.6
SonyAIBORobotSurface	3.4 ±0.0	45.0 ±20.7	**45.1 ±20.5**
SonyAIBORobotSurfaceII	2.5 ±0.0	**21.4 ±0.0**	**21.4 ±0.0**
TwoLeadECG	**1.4 ±0.0**	0.1 ±0.6	0.0 ±0.2
wafer	**0.0**	0.0	0.0
yoga	**0.5 ±0.0**	0.2 ±0.1	0.2 ±0.1

**Table 4:** NMI (in percents) of clusterings by kernel k-means and k-averages for datasets of 3 to 7 classes.

	spectral clustering	kernel k-means	k-averages
Beef	25.7 ±2.4	**35.5 ±2.8**	34.5 ±2.6
CBF	11.1 ±0.2	41.0 ±8.4	**41.0 ±7.0**
ChlorineConcentration	**3.9 ±0.0**	0.2 ±0.1	0.2 ±0.1
CinC_ECG_torso	**37.9 ±0.5**	24.2 ±1.5	24.6 ±1.6
DiatomSizeReduction	45.3 ±4.2	**79.8 ±6.3**	77.3 ±4.5
FaceFour	49.8 ±4.6	72.2 ±8.4	**74.9 ±6.3**
Haptics	3.0 ±0.5	**9.7 ±1.3**	9.4 ±1.2
InlineSkate	4.6 ±0.5	6.3 ±0.7	**6.4 ±0.7**
Lighting7	27.6 ±2.7	51.0 ±3.1	**51.3 ±1.5**
OSULeaf	22.9 ±0.8	22.9 ±2.2	**23.0 ±2.5**
OliveOil	10.6 ±2.2	**32.4 ±8.6**	30.6 ±7.8
StarLightCurves	54.3 ±0.0	**60.3 ±0.4**	60.3 ±0.0
Symbols	72.0 ±1.7	79.1 ±3.8	**79.5 ±1.6**
Trace	13.7 ±4.4	**54.5 ±5.1**	54.3 ±6.3
Two_Patterns	0.2 ±0.0	**10.1 ±10.0**	8.8 ±9.9
fish	18.5 ±1.5	34.9 ±1.4	**35.3 ±1.0**
synthetic_control	63.0 ±0.7	85.3 ±4.9	**89.5 ±0.8**

**Table 5:** NMI (in percents) of clusterings by kernel k-means and k-averages for datasets of 8 to 50 classes.

	spectral clustering	kernel k-means	k-averages
50words	46.9 ±1.0	69.6 ±0.8	**71.7 ±0.6**
Adiac	55.0 ±1.0	55.7 ±1.0	**58.9 ±0.6**
Cricket_X	19.2 ±1.1	26.1 ±1.8	**26.8 ±1.2**
Cricket_Y	22.8 ±1.3	33.0 ±1.5	**33.5 ±1.2**
Cricket_Z	19.2 ±1.3	25.5 ±2.2	**27.3 ±1.3**
FaceAll	36.4 ±1.2	**70.7 ±2.0**	68.2 ±2.3
FacesUCR	36.2 ±1.3	70.4 ±2.0	**71.5 ±1.6**
MALLAT	44.5 ±2.0	**90.3 ±4.6**	89.2 ±3.5
MedicalImages	19.2 ±1.3	29.7 ±1.7	**30.4 ±1.6**
SwedishLeaf	48.7 ±1.4	70.3 ±1.9	**70.8 ±1.4**
WordsSynonyms	31.9 ±1.0	50.9 ±1.2	**52.1 ±0.8**
uWaveGestureLibrary_X	24.4 ±0.6	46.0 ±1.2	**46.2 ±0.5**
uWaveGestureLibrary_Y	16.2 ±0.3	44.9 ±0.4	**45.0 ±0.2**
uWaveGestureLibrary_Z	23.2 ±0.6	**42.9 ±0.7**	42.6 ±0.5

For each experiment, the result of the best performing method is marked in bold. The Matlab and C implementations of the kernel k-means algorithm give exactly the same results in terms of NMI, thus only one column is used to display their performance.

A first observation is that the spectral clustering algorithm only performs favorably for 2 of the 43 dataset. Also, most of the bi-class problems (Table 3) do not seem to lend themselves well to this kind of approach: kernel k-means and k-averages produce quasi-identical results, poor in most cases. Concerning the medium numbers of classes (Table 4), k-averages performs best for 8 datasets out of 17. For the larger numbers of classes (Table 5), k-averages performs best for 11 datasets out of 14.

Considering the standard deviation over the several runs of the algorithm with different initialization is helpful to study the sensitivity of the algorithm to its initialization and thus its tendency to be stuck into local minima. For most datasets, the standard deviation of the k-averages algorithm is smaller than the one of the kernel k-means one and thus seems experimentally more robust.

### 7.5 Efficiency

Computation time displayed in Tables 6, 7, and 8 is the average duration over 100 runs on a single core with no parallelization capabilities. For every datasets, the spectral clustering approach is the more time consuming due to the diagonalization of the matrix which is of *O*(*N*^3^). For the kernel k-means algorithm, the C implementation is most of the time more efficient than the Matlab implementation. The k-average algorithm is more efficient for 46 datasets out of the 47 by close to an order of magnitude for the larger datasets.

**Table 6:** Computation time (in seconds) for several implementations of the evaluated clustering algorithms for datasets of 2 classes.

	Matlab		C	
	spectral clustering	kk-means	kk-means	k-averages
Coffee	0.02	0.00	**0.00**	0.00
ECG200	0.03	0.00	0.00	**0.00**
ECGFiveDays	0.13	0.10	0.06	**0.01**
Gun_Point	0.02	0.00	0.00	**0.00**
ItalyPowerDemand	0.20	0.07	0.05	**0.01**
Lighting2	0.02	0.00	0.00	**0.00**
MoteStrain	0.33	0.11	0.08	**0.01**
SonyAIBORobotSurface	0.09	0.02	0.01	**0.00**
SonyAIBORobotSurfaceII	0.09	0.01	0.02	**0.01**
TwoLeadECG	0.13	0.16	0.10	**0.02**
wafer	19.62	0.72	1.04	**0.39**
yoga	3.55	0.80	0.66	**0.10**

**Table 7:** Computation time (in seconds) for several implementations of the evaluated clustering algorithms for datasets of 3 to 7 classes.

	Matlab		C	
	spectral clustering	kk-means	kk-means	k-averages
Beef	0.02	0.00	0.00	**0.00**
CBF	0.17	0.08	0.05	**0.01**
ChlorineConcentration	7.15	2.74	2.09	**0.23**
CinC_ECG_torso	0.55	0.31	0.16	**0.04**
DiatomSizeReduction	0.04	0.01	0.00	**0.00**
FaceFour	0.02	0.00	0.00	**0.00**
Haptics	0.07	0.04	0.01	**0.01**
InlineSkate	0.12	0.08	0.03	**0.01**
Lighting7	0.04	0.01	0.00	**0.00**
OSULeaf	0.08	0.04	0.01	**0.00**
OliveOil	0.03	0.00	0.00	**0.00**
StarLightCurves	34.16	4.69	6.21	**0.91**
Symbols	0.26	0.15	0.06	**0.02**
Trace	0.02	0.01	0.00	**0.00**
Two_Patterns	11.99	3.99	3.71	**0.40**
fish	0.06	0.02	0.01	**0.00**
synthetic_control	0.09	0.03	0.01	**0.01**

**Table 8:** Computation time (in seconds) for several implementations of the evaluated clustering algorithms for datasets of 8 to 50 classes.

	Matlab		C	
	spectral clustering	kk-means	kk-means	k-averages
50words	0.89	0.19	0.05	**0.03**
Adiac	0.49	0.16	0.05	**0.03**
Cricket_X	0.24	0.10	0.05	**0.02**
Cricket_Y	0.22	0.09	0.05	**0.02**
Cricket_Z	0.22	0.09	0.04	**0.02**
FaceAll	2.08	0.78	0.40	**0.11**
FacesUCR	2.21	0.75	0.41	**0.12**
MALLAT	2.23	0.47	0.29	**0.12**
MedicalImages	0.42	0.24	0.12	**0.04**
SwedishLeaf	0.56	0.22	0.11	**0.04**
WordsSynonyms	0.44	0.16	0.08	**0.03**
uWaveGestureLibrary_X	8.60	5.15	3.82	**0.44**
uWaveGestureLibrary_Y	8.94	4.48	2.65	**0.45**
uWaveGestureLibrary_Z	8.53	4.00	2.89	**0.47**

### 7.6 Overall results

To conclude on the performance of the evaluated algorithms on real datasets, Table 9 displays the NMI and computation time averaged over the 47 datasets. The k-averages method marginally improve the clustering accuracy compared to the kernel k-means approach by using less time to compute.

**Table 9:** Performances averaged over the 47 datasets. The NMI is expressed in percents and the computation time in seconds.

	Matlab		C	
	spectral clustering	kk-means	kk-means	k-averages
nmi (%)	22.1	36.6	36.6	**36.8**
computation time	2.678	0.723	0.590	**0.096**

## 8 Conclusion

We have presented k-averages, an iterative flat clustering algorithm that operates on arbitrary similarity matrices by explicitly and directly aiming to optimize the average intra-class similarity. Having established the mathematical foundation of our proposal, including guaranteed convergence, we have thoroughly compared it with widely used standard methods: the kernel k-means and the spectral clustering techniques. We show that the k-averages algorithm converges much faster (20 times faster under ordinary conditions) and leads to equivalent or better clustering results for the task of clustering both synthetic data and realistic times series taken from a wide variety of sources, while also being more computationally efficient and more sparing in memory use.
